# Sporadic renal angiomyolipoma in a patient with Birt-Hogg-Dubé: chaperones in pathogenesis

**DOI:** 10.18632/oncotarget.25164

**Published:** 2018-04-24

**Authors:** Rebecca A. Sager, Mark R. Woodford, Oleg Shapiro, Mehdi Mollapour, Gennady Bratslavsky

**Affiliations:** ^1^ Department of Urology, SUNY Upstate Medical University, Syracuse, NY, USA; ^2^ Upstate Cancer Center, SUNY Upstate Medical University, Syracuse, NY, USA; ^3^ Department of Biochemistry and Molecular Biology, SUNY Upstate Medical University, Syracuse, NY, USA

**Keywords:** Birt-Hogg-Dubé (BHD), FLCN, tuberous sclerosis complex (TSC), renal angiomyolipoma, TSC1 (Hamartin)

## Abstract

Birt-Hogg-Dubé (BHD) is an autosomal dominant genetic syndrome caused by germline mutations in the *FLCN* gene that predisposes patients to develop renal tumors. Renal angiomyolipoma (AML) is not a renal tumor sub-type associated with BHD. AML is, however, a common phenotypic manifestation of Tuberous Sclerosis Complex (TSC) syndrome caused by mutations in either the *TSC1* or *TSC2* tumor suppressor genes. Previous case reports of renal AML in patients with BHD have speculated on the molecular and clinical overlap of these two syndromes as a result of described involvement of the gene products in the mTOR pathway. Our recent work provided a new molecular link between these two syndromes by identifying FLCN and Tsc2 as clients of the molecular chaperone Hsp90. Folliculin interacting proteins FNIP1/2 and Tsc1 are important for FLCN and Tsc2 stability as new Hsp90 co-chaperones. Here we present a case of sporadic AML as a result of somatic Tsc1/2 loss in a patient with BHD. We further demonstrate that FNIP1 and Tsc1 are capable of compensating for each other in the chaperoning of mutated FLCN tumor suppressor. Our findings demonstrate interconnectivity and compensatory mechanisms between the BHD and TSC pathways.

## INTRODUCTION

Birt-Hogg-Dubé (BHD) is an autosomal dominant genetic syndrome caused by germline mutations in the *FLCN* gene on chromosome 17p11.2, which encodes the protein Folliculin (FLCN) [1–4]. Phenotypic manifestations of BHD include cutaneous fibrofolliculomas or trichodiscomas, pulmonary cysts and spontaneous pneumothorax, and renal tumors [5, 6]. Renal tumors occur in up to 1/3 of BHD patients; the most common histologic subtype is hybrid oncocytic, followed by chromophobe renal cell carcinoma (chRCC), clear cell RCC, and renal oncocytoma [7–9]. Renal angiomyolipoma (AML) is not, however, a described renal tumor sub-type associated with BHD, but is instead a common manifestation of Tuberous Sclerosis Complex (TSC) syndrome.

TSC is also an autosomal dominant genetic syndrome and is caused by germline mutations in either the *TSC1* or *TSC2* tumor suppressor genes, which encode the Tsc1 and Tsc2 proteins, also known as hamartin and tuberin [10–12]. In addition to neural associations that include epilepsy, subependymal giant cell astrocytomas (SEGA), intellectual disability, and autism, TSC is also characterized by cutaneous, pulmonary, and renal manifestations, similarly to BHD [10]. These include cutaneous facial angiofibromas, pulmonary lymphangioleiomyomatosis (LAM), and renal AMLs.

A number of case reports have reported overlapping phenotypic manifestations of BHD and TSC, including three cases of renal AML in patients with BHD [13–17]. Authors have then speculated on the overlap of the spectrum of these two syndromes due to similarities in phenotype and putative involvement of the implicated genes in the mTOR pathway. Canonically, Tsc2 exerts an inhibitory role on the mTORC1 complex via its GAP activity towards the small GTPase Rheb, whereby loss of Tsc2 leads to mTOR pathway hyperactivity [18–20]. Tsc1 was long known to be important for the stability of Tsc2, preventing its ubiquitination and degradation [21, 22]. Recently, we reported that Tsc2 is a client of the molecular chaperone heat shock protein-90 (Hsp90) and depends on Hsp90 activity for its stability [23]. In contrast, Tsc1 is a co-chaperone of Hsp90 and is important for the chaperoning of client proteins, including Tsc2 [23]. FLCN has also been tied to the mTOR pathway because FLCN-deficient animal models showed alterations of mTOR activity [24–27]. Two folliculin-interacting proteins, FNIP1 and FNIP2, were identified to be important for FLCN stability [28, 29]. We have also identified FLCN as a new client of Hsp90 and FNIP1 and FNIP2 as new Hsp90 co-chaperones [30].

Hsp90 is an essential molecular chaperone in eukaryotes that is required for the stability and activation of a number of client proteins, including Tsc2 and FLCN, to maintain proteostasis [31]. Hsp90 function is coupled to its ATPase activity, which consequently promotes Hsp90 to undergo an ordered series of conformational changes known as the chaperone cycle. The Hsp90 chaperone cycle is regulated by a group of proteins referred to as co-chaperones, including FNIP1, FNIP2, and Tsc1. The stability of co-chaperones does not depend on the chaperone function of Hsp90; however, they directly bind to Hsp90 and modulate its ATPase activity. Consequently, this facilitates the loading of client proteins to Hsp90 and ultimately the chaperoning of these clients [31, 32].

Here, we report a case of renal AML in a patient with BHD. Molecular analysis of the AML and adjacent normal kidney tissue from this patient demonstrates that this tumor is a sporadic renal AML caused by somatic loss of Tsc1/2 protein. Further experiments utilizing a FLCN construct harboring the germline mutation of this patient suggest that there may be some ability of FNIP1 and Tsc1 to compensate for one another in the chaperoning of the FLCN and Tsc2 tumor suppressors.

## CASE REPORT

A 53-year-old woman presented to our Urology clinic for consultation for her BHD syndrome. She was diagnosed at age 51 at another institution after multiple spontaneous pneumothoraces requiring pleurodesis. At the time, she was noted to have multiple lung cysts, multiple small (centimeter and sub-centimeter) renal cysts, and a 2.8 cm right kidney AML on CT scan. At age 43, she had a right parotid gland lesion excised, which was shown to be a parotid oncocytoma. Parotid oncocytomas have been previously reported in the setting of BHD, but it has not yet been shown to be a true phenotypic feature [33–38]. Her sister, oldest son, and niece tested positive for BHD; her younger son and father were negative, and her nephew was not tested at that time. There was no known family history of kidney cancer. Her mother passed away at age 69 of lung disease. Her maternal grandmother had polycystic kidneys, and a paternal aunt passed away of an unspecified kidney disease. There were no other known symptomatic family members at the time of diagnosis.

Genetic testing revealed a heterozygous mutation in *FLCN* (c.1379_1380delTC or p.Leu460GlnsX25) in the patient, and this same mutation was later identified in multiple family members. This mutation has been reported previously; deletion of two nucleotides in exon 12 of the *FLCN* gene causes a frameshift mutation, changing L460 to glutamine and creating a premature stop codon at position 25 of the new reading frame [34]. Due to her renal AML seen on imaging, which is common in TSC but not a characteristic of BHD, genetic testing for germline mutations of *TSC1* and *TSC2* was also carried out, however no mutations in either gene were identified.

**Figure 1:**
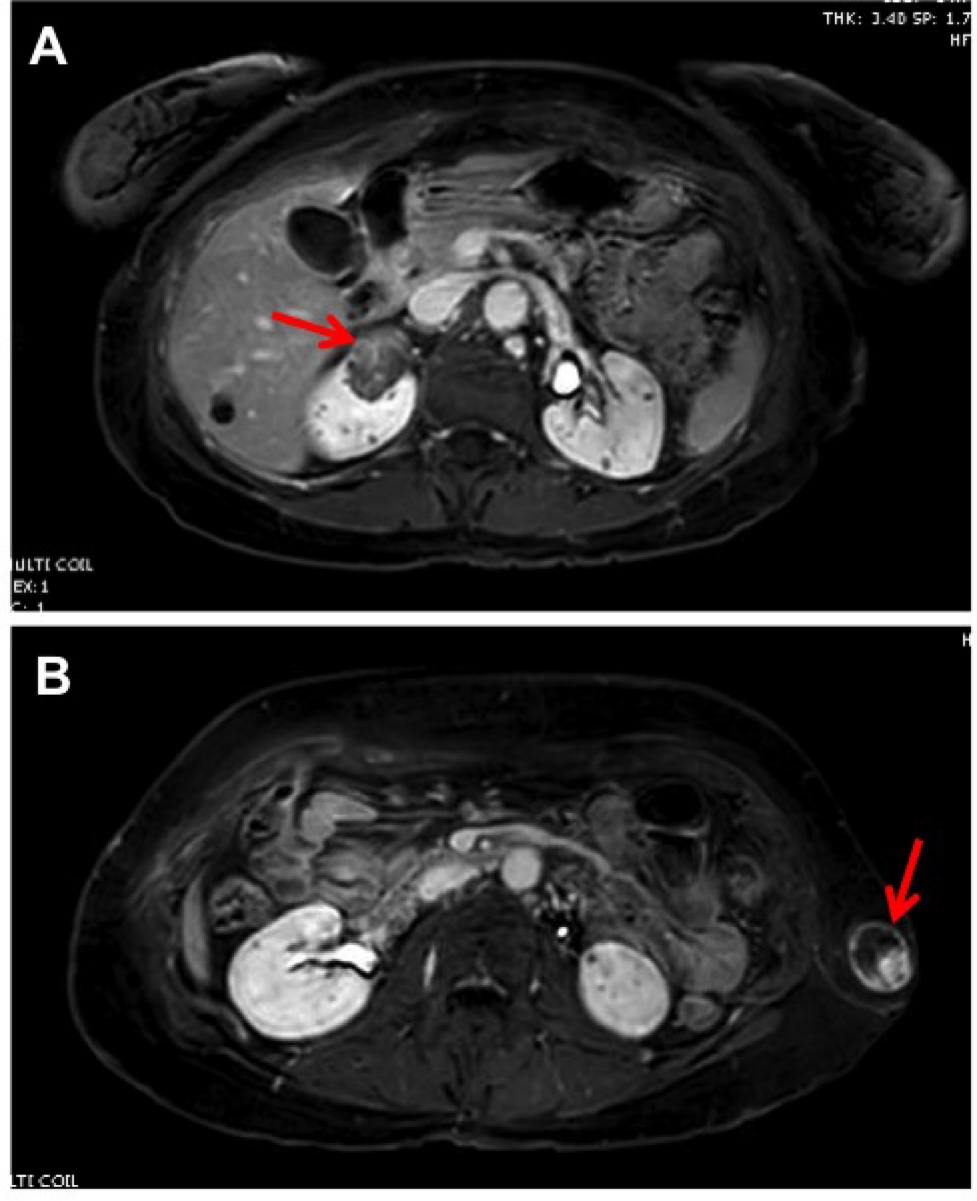
Magnetic resonance imaging (MRI) of the abdomen and pelvis (T1 with gadolinium) demonstrating (**A**), solid renal mass with radiographic evidence of fat in the upper pole of the right kidney and (**B**), left flank mass.

**Figure 2:**
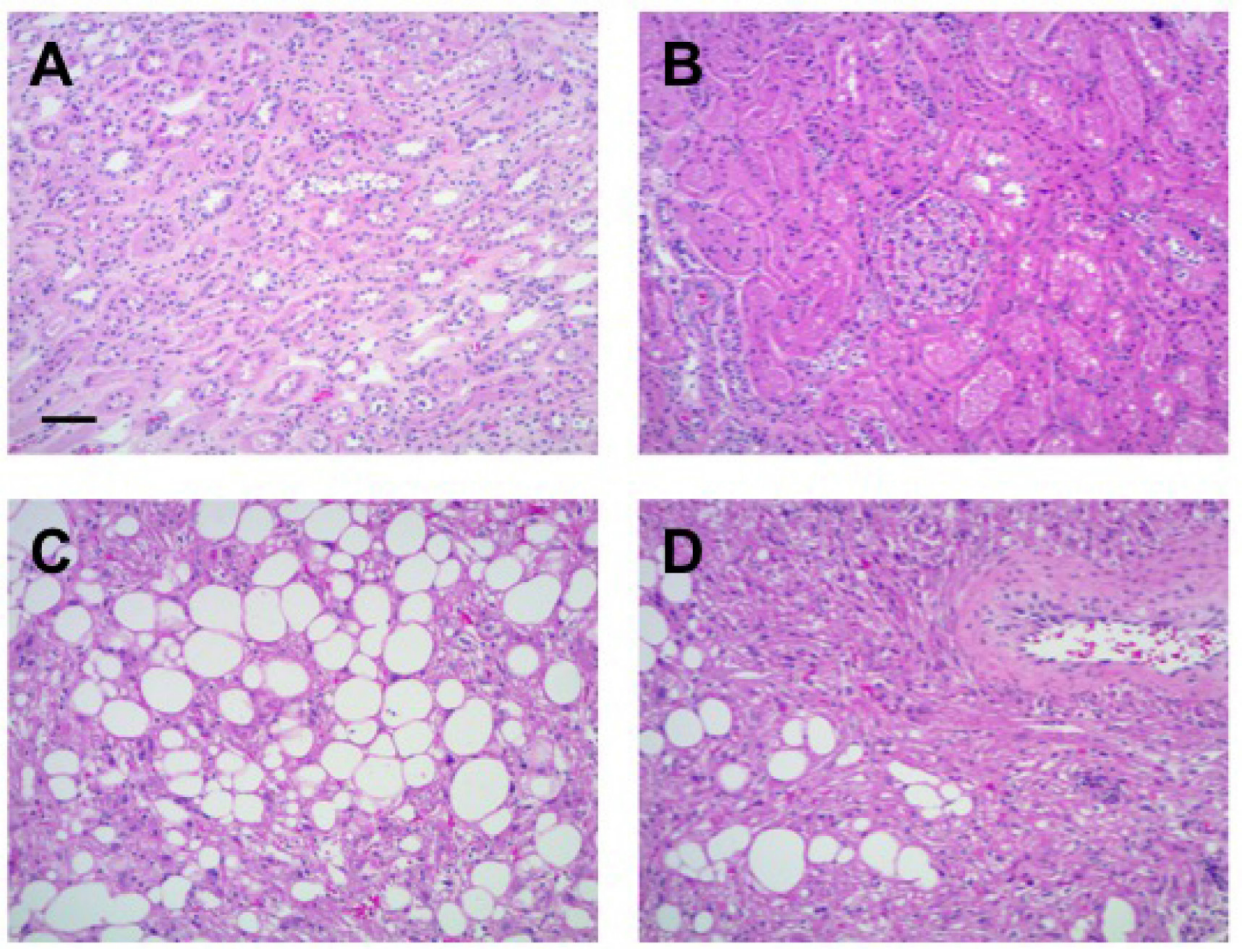
Hematoxylin and eosin (H&E) stained sections of adjacent normal kidney far (**A**) and close (**B**) to the tumor. Tumor histology (**C** and **D**) is consistent with renal AML. Scale bar = 50 µm.

A few months prior to renal surgery, our patient had a right-sided pleurectomy and pleurodesis after another spontaneous pneumothorax. We performed a right partial nephrectomy to remove her renal mass. Decision to pursue surgery came after follow-up MRI demonstrated interval growth of her renal lesion to become over 3 cm (Figure 1A). The patient had also recently lost her 26-year-old nephew, with confirmed germline *FLCN* mutation, to metastatic renal cancer. Interval growth of the lesion, anxiety of the patient, and concern for a potential association and co-existence of clear cell renal cell carcinoma with and within the observed AML contributed to the patient’s request to proceed with robotic assisted partial nephrectomy. Removal of a breast cyst and left flank mass that the patient had for years (Figure 1B) were also coordinated at the time of partial nephrectomy. Final pathology revealed that the renal mass was an angiomyolipoma (Figure 2), the breast cyst was an epidermal inclusion cyst, and the left flank mass was a Schwannoma.

## RESULTS

### Identification of a sporadic AML in a patient with BHD

**Figure 3:**
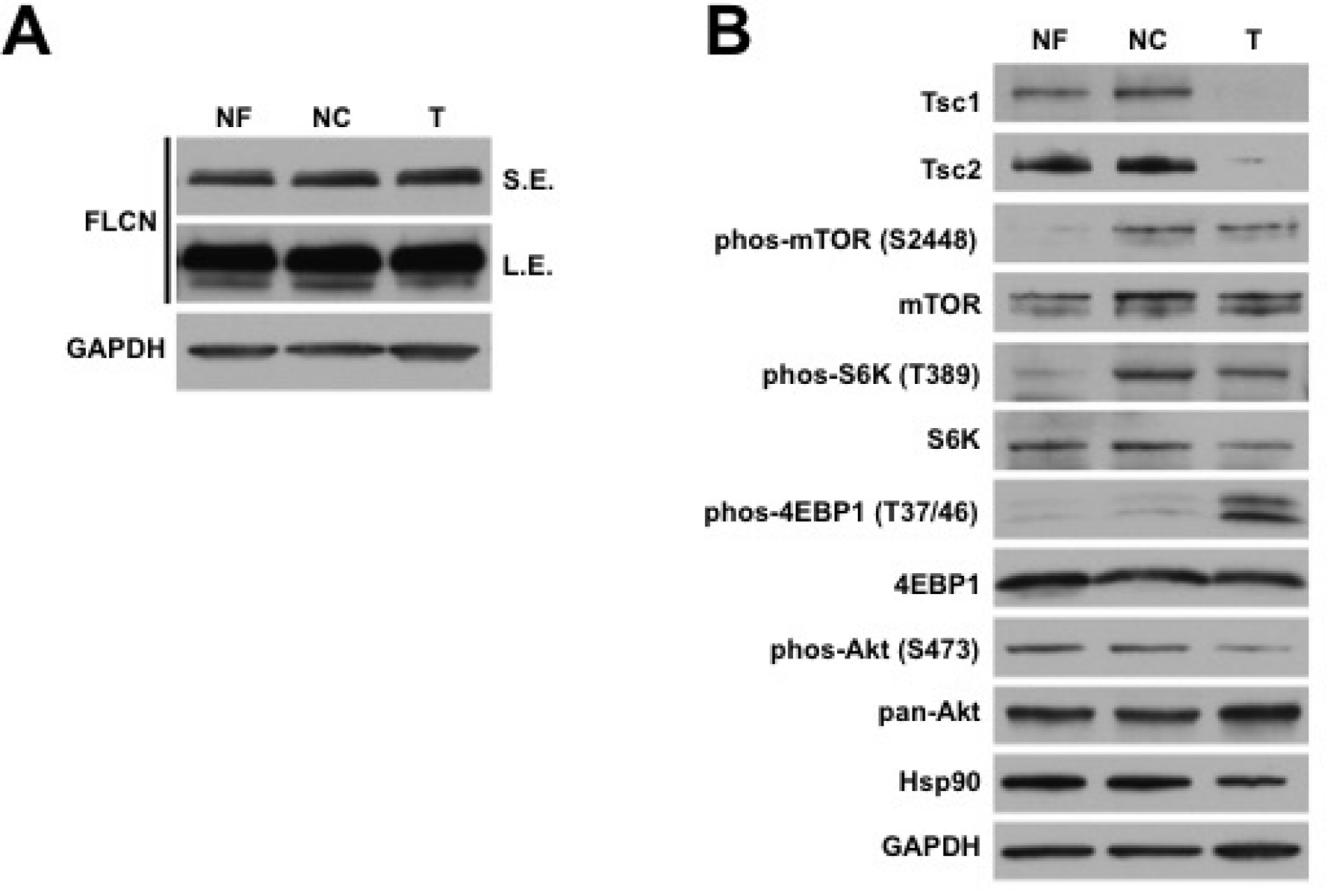
Sporadic renal AML demonstrates somatic loss of Tsc1/2 expression (**A**) Protein was extracted from adjacent normal far (NF) and close (NC) kidney and tumor (T). Expression of FLCN was examined by immunoblotting. GAPDH was used as a loading control. Short (SE) and long (LE) exposure of the radiographic film. (**B**) Protein was extracted from adjacent normal far (NF) and close (NC) kidney and tumor (T). Expression of Tsc1/2 and mTOR pathway components was examined by immunoblotting. GAPDH was used as a loading control.

Prior to surgery, the patient had given informed consent on an institutional IRB-approved protocol granting research access to her renal tumor and associated normal kidney tissue. After partial nephrectomy with margin of healthy parenchyma and additional biopsy of the uninvolved parenchyma away from the resection site was performed, tumor and adjacent close and far normal kidney tissue were dissected into 5 mm^3^ pieces followed by protein extraction and immunoblotting analysis. Our data demonstrated that the renal AML had equal FLCN expression to adjacent normal kidney (Figure 3A). Long exposure of the radiographic film also demonstrated a band of weaker intensity below the full-length FLCN, which we believed was the unstable protein product of the patient’s mutated *FLCN* allele (Figure 3A). We next examined the levels of Tsc1 and Tsc2 protein in the AML and found that both of them were absent (Figure 3B). Consistent with the loss of Tsc1/2, there were elevated levels of phosphorylated mTOR (S2448), S6K (T389), and 4EBP1 (T37/46) (Figure 3B). Interestingly, the phosphorylated mTOR and S6K were also elevated in the normal close tissue, which did not exhibit loss of Tsc1/2 protein expression (Figure 3B). This did not hold true for phospho-4EBP1, which was overexpressed in tumor only. Additionally, there was a decrease in phospho-S473-Akt. Taken together, our data suggests that this tumor is a sporadic renal AML as a result of somatic loss of Tsc1/2 in the setting of BHD syndrome.

### FLCN-L460QsX25 mutant interacts with and is stabilized by Tsc1

**Figure 4:**
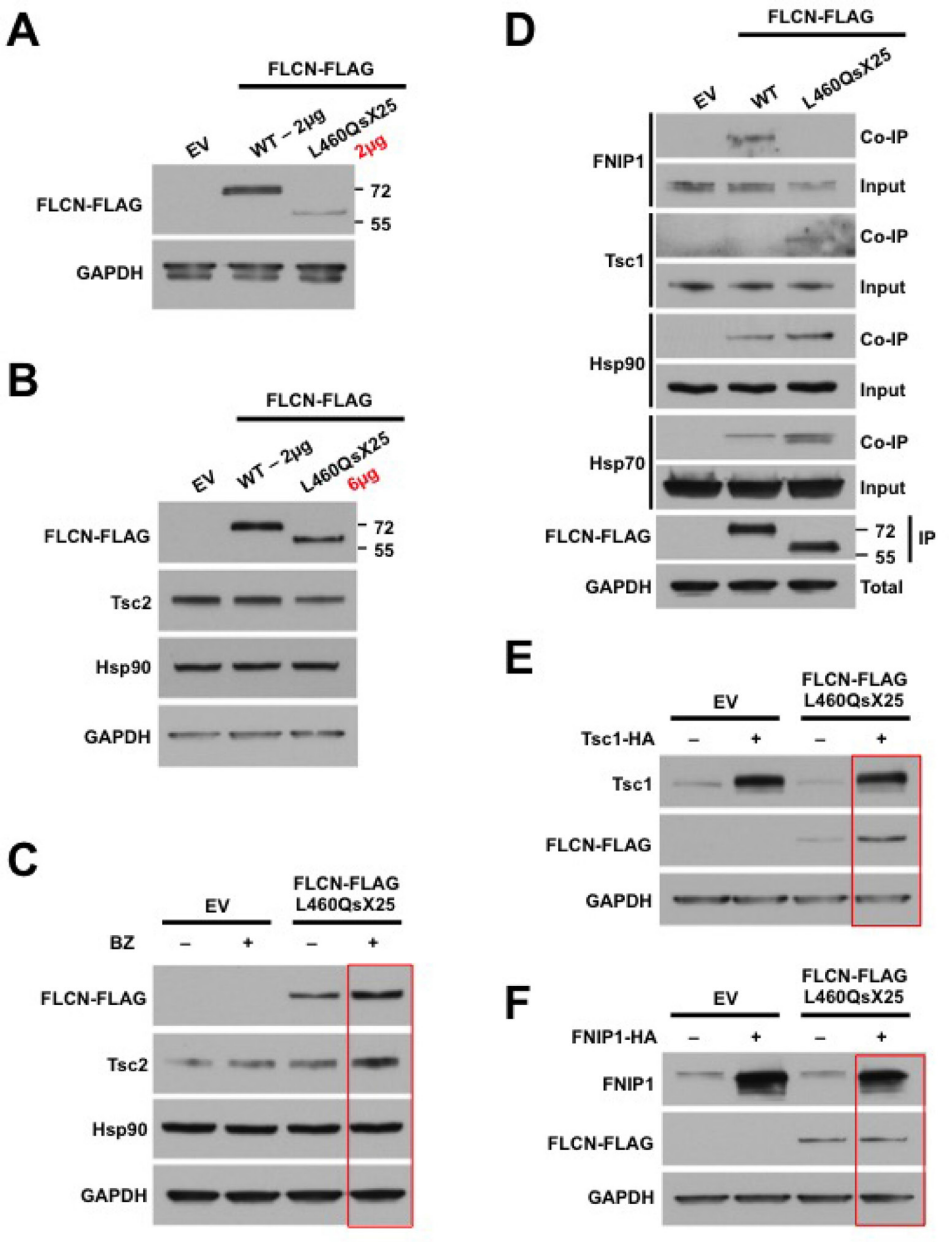
FLCN-L460QsX25 mutant interacts with and is stabilized by Tsc1 (**A**) HEK293 cells were transiently transfected with 2 µg of either WT or L460QsX25 mutated FLCN-FLAG. Expression was assessed by immunoblotting. Empty vector (EV) was used as a control. (**B**) HEK293 cells were transiently transfected with 2 µg WT or 6 µg L460QsX25 mutated FLCN-FLAG. Expression of FLCN-FLAG and Tsc2 was assessed by immunoblotting. EV was used as a control. (**C**) HEK293 cells were transiently transfected with 1 µg of EV or FLCN-L460QsX25-FLAG for 24 hr and then treated with 200 nM proteasome inhibitor bortezomib (BZ) for 4 hr prior to protein extraction. Stability of FLCN-FLAG and Tsc2 was assessed by immunoblotting. (**D**) WT and L460QsX25 mutated FLCN-FLAG were transiently expressed and immunoprecipitated from HEK293 cells. Co-immunoprecipitation (Co-IP) of endogenous FNIP1, Tsc1, Hsp90, and Hsp70 was assessed by immunoblotting. EV was used as a control. (**E**) HEK293 cells were transiently transfected with either EV or FLCN-L460QsX25-FLAG with or without co-transfection of Tsc1-HA. Those cells without co-expression of Tsc1-HA instead had additional EV co-transfected. Stability of FLCN-FLAG was assessed by immunoblotting. Overexpression of Tsc1 was demonstrated by probing the blot with an anti-Tsc1 antibody. GAPDH was used as a loading control. (**F**) HEK293 cells were transiently transfected with either EV or FLCN-L460QsX25-FLAG with or without co-transfection of FNIP1-HA. Those cells without co-expression of FNIP1-HA instead had additional EV co-transfected. Stability of FLCN-FLAG was assessed by immunoblotting. Overexpression of FNIP1 was demonstrated by probing the blot with an anti-FNIP1 antibody. GAPDH was used as a loading control.

Pathogenic mutations of *FLCN* that result in premature truncation of the protein have previously been reported to be unstable and therefore non-functional [3]. Additionally, FLCN interacts with FNIP1 via its C-terminus [28]; FLCN truncation would prevent FNIP1 from loading it to Hsp90 for chaperoning [30]. We therefore used site-directed mutagenesis to generate a FLCN construct harboring this patient’s germline *FLCN* mutation, FLCN-FLAG-L460QsX25, to examine the effects of this mutation on FLCN stability and chaperoning. This mutation causes a shift in the reading frame followed by a premature stop codon, truncating the FLCN protein. Consistent with this, the mutant FLCN-L460QsX25 expression was weaker than the WT after transfection of equal amounts of plasmid DNA (2 µg) for FLCN-FLAG-WT and FLCN-FLAG-L460QsX25 into HEK293 cells (Figure 4A). However, overexpression of this mutant (i.e. transfection with 6 µg of DNA instead of 2 µg) equilibrated its expression to that of FLCN-FLAG-WT (Figure 4B). The level of Tsc2 was also decreased in the sample with overexpressed L460QsX25 (Figure 4B). Transfection of only 1 µg of mutant FLCN for 24 hr followed by treatment with the proteasome inhibitor bortezomib for 4 hr was able to stabilize the expression of FLCN-L460QsX25 (Figure 4C). Treatment with bortezomib in the presence of the FLCN mutant also led to a greater accumulation of Tsc2 than in EV transfected cells, possibly suggesting that Tsc2 is undergoing increased turnover in the presence of FLCN-L460QsX25 (Figure 4C).

We next immunoprecipitated WT FLCN-FLAG and FLCN-FLAG-L460QsX25 in order to examine interaction of the mutant with the Hsp90 chaperone machinery. As expected, C-terminal truncation in this FLCN mutant abrogated its interaction with FNIP1 (Figure 4D) [28]. The mutant FLCN still interacted with Hsp90 as well as Hsp70, which was unexpected because our previous work has shown that loading and interaction of FLCN with Hsp90 requires the FNIP co-chaperones [30]. Additionally, the mutant FLCN co-immunoprecipitated more Tsc1 than WT-FLCN (Figure 4D). We therefore next examined the ability of overexpression of both Tsc1 and FNIP1 to stabilize FLCN-FLAG-L460QsX25 expression. Overexpression of Tsc1-HA (Figure 4E) but not FNIP1-HA (Figure 4F) was able to stabilize the expression of FLCN-FLAG-L460QsX25. Taken together, these data suggest that Tsc1 co-chaperone may be able to partially compensate for FNIP1 co-chaperone in the chaperoning of the FLCN-L460QsX25 mutant.

## DISCUSSION

BHD syndrome is characterized by cutaneous, pulmonary, and renal manifestations [3]. Renal tumors in BHD are most commonly of hybrid oncocytic or chromophobe histology, and renal AML has not been associated with BHD. Here we present a case of renal AML, a kidney neoplasm typically associated with TSC syndrome, in a patient with BHD. Previous case reports of AML in the setting of BHD in the literature posit on a phenotypic overlap between TSC and BHD, suggesting that renal AML may be a manifestation of BHD in those affected patients [13, 15]. However, studies on renal AML have demonstrated that both TSC-associated and sporadic renal AMLs exhibit mutations and/or LOH in *TSC1* or *TSC2* [39–41]. Loss of Tsc1/2 is considered the sufficient driver event for tumor development, and these tumors exhibit no other common genetic events. Loss of Tsc1/2 leads to activation of mTOR and its downstream signaling components, such as increased phosphorylation of S6K, in both TSC-associated and sporadic AMLs compared to normal kidney [42, 43].

In this case we demonstrate that this tumor is a sporadic renal AML as evidenced by somatic loss of Tsc1/2 protein in the tumor tissue compared to adjacent normal kidney without loss of FLCN protein expression (Figure 3). Consistent with this, the AML tissue shows increased phosphorylation of mTOR and its downstream targets, S6K and 4EBP1, as a result of loss of the inhibitory signal from Tsc2 on mTORC1 signaling. Interestingly, the phosphorylated mTOR and S6K were also elevated in the normal close tissue, which did not exhibit loss of Tsc1/2 protein expression. This potentially represents a “field defect” of normal parenchyma adjacent to the tumor compared to the normal renal parenchyma far from the tumor.

Sequencing of *TSC1* and *TSC2* in whole blood from our patient did not identify any mutations, suggesting that this patient likely does not have TSC in addition to BHD syndrome. Sporadic cases of AML have been shown to be nearly exclusively due to loss of Tsc2 [40, 41, 43]. We attempted to sequence the normal kidney and tumor tissue to identify possible somatic mutations in *TSC1* or *TSC2*; however, unfortunately we were unable to obtain high quality sequence information. The absence of both Tsc1 and Tsc2 protein expression in the tumor, however, suggests loss of Tsc1 is driving this neoplasm because Tsc2 is known to depend on Tsc1 for stability, but Tsc1 stability does not depend on the presence Tsc2.

This case was of particular interest to us because our recent work has described new roles of FLCN and its interacting proteins FNIP1/2 as well as the Tsc1 and Tsc2 proteins with the Hsp90 molecular chaperone machinery. We identified that both FLCN and Tsc2 are clients of Hsp90 and that FNIP1 and Tsc1 are Hsp90 co-chaperones that assist in the chaperoning of not only FLCN and Tsc2 but also other Hsp90 client proteins [23, 30]. The identification of FNIP1 and Tsc1 as co-chaperones has created a new class of large (both are >130 kDa) co-chaperones of Hsp90, and they share a number of similarities in this new role. Both bind the middle domain of Hsp90 through their C-termini, decelerate Hsp90 ATPase activity, and compete with accelerating co-chaperone Aha1 for binding to Hsp90. They also both affect chaperoning of both kinase and non-kinase clients and help facilitate interaction of their respective tumor suppressor clients, FLCN and Tsc2, with Hsp90. The many similarities of FNIP1 and Tsc1 as Hsp90 co-chaperones, coupled with the similar clinical phenotypes of BHD and TSC syndromes and unique identification of sporadic AML in this patient with BHD prompted us to investigate the chaperoning of the mutant FLCN protein that results from the germline mutation of this patient.

To examine the stability and chaperoning of this FLCN mutant, we generated a construct containing the germline mutation present in this patient, FLCN-FLAG-L460QsX25. As mentioned above, this frameshift mutation in exon 12 causes a shift in the reading frame at position L460 and a premature stop codon at position 25 of the new reading frame. Transient transfection of FLCN-FLAG-L460QsX25 demonstrated that it was less stable than WT FLCN, as expected from a C-terminal truncation that abrogates its binding to FNIP1 and subsequently prevents FNIP1 from loading FLCN to Hsp90. Interestingly, this mutant bound as much Hsp90 as the WT and bound more Tsc1. Furthermore, overexpression of Tsc1-HA, but not FNIP1-HA, was able to stabilize the expression of FLCN-FLAG-L460QsX25. This suggests that perhaps Tsc1 co-chaperone is partially compensating for FNIP1 in chaperoning of the mutant FLCN-L460QsX25. Additionally, in cells with increased overexpression of FLCN-FLAG-L460QsX25 the Tsc2 level was decreased, and treatment with bortezomib in the presence of the mutant FLCN led to a greater increase in Tsc2 than in the EV transfected, suggesting an increased turnover of Tsc2. One possible explanation for all of this taken together is that Tsc1 can assist in the chaperoning of FLCN-FLAG-L460QsX25 when the mutant FLCN is overexpressed by transient transfection; Tsc1 is therefore less available for chaperoning of Tsc2 causing Tsc2 levels to decrease. These data raise the idea that these two large co-chaperones, FNIP1 and Tsc1, may possess some ability to compensate for one another in the chaperoning of tumor suppressors and, more specifically, mutated tumor suppressors.

What remains to be explored, however, is whether there is a causal link between BHD syndrome and development of sporadic AML. Other reports of renal AML in BHD patients did not have tissue to demonstrate whether the AML exhibited loss of FLCN or Tsc1/2 expression. They were, however, all single lesions while kidney tumors associated with genetic syndromes tend to be multifocal and bilateral, so it is possible that these are all cases of sporadic AML caused by somatic Tsc1/2 loss in patients with BHD. Currently, we are unable to determine whether patients with BHD are more likely than the general population to develop sporadic renal AML because a true prevalence for neither AML nor BHD is known. To our knowledge this is the fourth reported case of AML in the setting of BHD in the literature. Due to the rarity of BHD, these four cases may suggest a pathophysiologic link between BHD syndrome and likelihood of AML development. This is the first report to provide mechanistic insight into how AML pathogenesis could be unique in the setting of BHD syndrome. Our recent identification of the involvement of the Hsp90 chaperone machinery with the proteins implicated in BHD and TSC as well as the case report and molecular characterization presented here suggest there may be more complexity to the clinical and molecular overlap between these syndromes than previously appreciated. Examination of new functions for both Tsc1 and FLCN in particular is warranted.

## MATERIALS AND METHODS

### Mammalian cell culture

Human embryonic kidney (HEK293) cells were acquired from the American Type Culture Collection (ATCC) and grown in Dulbecco’s Modified Eagle Medium (DMEM, Sigma–Aldrich). Their media was supplemented with 10% fetal bovine serum (FBS, Sigma–Aldrich), and they were grown in a CellQ incubator (Panasonic Healthcare) at 37° C in an atmosphere containing 5% CO_2_.

### Plasmids

Both FLCN-FLAG and FNIP1-HA were used previously [30]. pcDNA3–Tsc1–HA was purchased from Addgene and was used previously [23]. FLCN-FLAG-L460QsX25 was generated by site-directed mutagenesis using the following primers: FLCN-L460QsX25-F – GATGACCAGTCTCAGCAAGTACGAG; and FLCN-L460QsX25-R – CTCGTACTTGCTGAGACTGGTCATC. Mutations were checked by DNA sequencing.

### Analysis of human kidney tumor

Tumor and adjacent normal tissues of the patient were obtained with written informed consent from the Department of Urology at SUNY Upstate Medical University. At the time of partial nephrectomy, which was done with <10 minutes of renal ischemia, tissue was dissected into approximately 5 mm^3^ pieces and protein was extracted and quantified as previously described [44]. The tissues were also formalin-fixed, paraffin embedded, and stained with H&E.

### Protein extraction, immunoprecipitation, and immunoblotting

Protein extraction from mammalian cells was carried out using methods previously described [44]. For immunoprecipitation, protein lysates were incubated with anti-FLAG M2 Affinity Gel agarose (Sigma) for 2 hr at 4° C. Immunopellets were washed 4 times with fresh lysis buffer (20 mM HEPES (pH7.0), 100 mM NaCl, 1 mM MgCl_2_, 0.1% NP40, protease inhibitor cocktail (Roche), and PhosSTOP (Roche)) and eluted with 5× Laemmli buffer. Precipitated proteins were separated by SDS-PAGE and transferred to nitrocellulose membranes. Co-immunoprecipitated proteins were detected by immunoblotting with indicated dilutions of antibodies. Primary antibodies recognizing FLAG 1:8000 (ThermoFisher Scientific; PA1-984B), Hsp90-835-16F1 1:8000 (ENZO Life Sciences; ADI-SPA-835), GAPDH 1:10,000 (ENZO Life Sciences; ADI-CSA-335), Hsp70 1:40,000 (StressMarq; SPC-103), FLCN 1:4000 (Cell Signaling Technologies (CST); 3697), Tsc1 1:1000 (CST; 4906), Tsc2 1:1000 (CST; 3990), phos-mTOR 1:1000 (S2448; CST; 5536), mTOR 1:1000 (CST; 2983), phos-S6K 1:6000 (T389; CST; 9234), phos-4EBP1 1:2000 (T37/46; CST; 2855), 4EBP1 1:2000 (CST; 9644), phos-Akt 1:2000 (S473; CST; 4060); Akt 1:2000 (CST; 9727), S6K 1:6000 (SantaCruz Biotechnology; sc-8418) were used for immunoblotting. Secondary antibodies raised against mouse, rabbit, and rat (Cell Signaling Technologies) were used at 1:4000 dilution.

## Abbreviations

AML: angiomyolipoma
BHD: Birt-Hogg-Dubé
CT: computed tomography
FLCN: Folliculin
FNIP1: Folliculin-interacting protein-1
GAP: GTPase activating protein
Hsp90: Heat shock protein-90
IRB: institutional review board
LAM: lymphangioleiomyomatosis
MRI: magnetic resonance imaging
mTOR: mechanistic target of rapamycin
RCC: renal cell carcinoma
SEGA: subependymal giant cell astrocytoma
TSC: Tuberous Sclerosis Complex
